# Cognitive vulnerabilities in adolescents with suicidal risk

**DOI:** 10.1192/j.eurpsy.2026.10574

**Published:** 2026-07-31

**Authors:** J. Gjino, M. Orlandi, M. Di Girolamo, R. Invernizzi, M. M. Mensi

**Affiliations:** 1 Department of Brain and Behavioral Sciences, University of Pavia; 2 Child Neurology and Psychiatry Unit, IRCSS Mondino Foundation, Pavia; 3 Department of Psychology, University of Turin, Turin; 4 Child Neurology and Psychiatry, ASST Lecco, Lecco, Italy

## Abstract

**Introduction:**

Suicide is a leading cause of death among adolescents, representing a major global health concern (World Health Organization, 2024). While psychosocial risk factors are widely studied, less is known about the role of cognitive functioning. Evidence indicates that poor cognitive performances, particularly in executive functions (EF) and memory, are linked to suicidal concern (SC)(Burton et al., 2011). Research has shown deficits in attention, cognitive flexibility, working memory and inhibitory control among individuals with SC (Bredemeier & Miller, 2015; Keilp et al., 2013; Richard-Devantoy et al., 2023). However, studies in adolescents using standardized intelligence measures remain limited.

**Objectives:**

This study aimed to compare cognitive functioning between adolescents with and without SC, examine associations between cognitive indices and suicidal ideation/behaviors and explore the role of cognitive vulnerabilities in risk stratification.

**Methods:**

In this cross-sectional study, 261 adolescents aged 12–18 admitted to Child Neuropsychiatry Services in Northern Italy (2020–2024) were included after excluding cases with cognitive impairment (IQ ≤ 70), insufficient knowledge of Italian, or no written consent. SC was assessed with the Columbia-Suicide Severity Rating Scale (C-SSRS), and cognitive functioning with WISC-IV or WAIS-IV. Participants were classified as SC (n = 200) or non-suicidal concern (NSC; n = 61). Independent t-tests compared Wechsler scales indices; Pearson’s correlations examined associations between cognitive scores and C-SSRS dimensions.

**Results:**

Adolescents with SC were more often female and showed poorer social relationships and higher rates of NSSI. Our analysis showed lower total IQ, Working Memory Index (WMI), and Perceptual Reasoning Index (PRI) in the SC group compared to NSC, while no differences emerged in Verbal Comprehension or Processing Speed. Correlational analyses showed that reduced WMI and PRI were consistently linked to greater severity, frequency, and behavioral expression of suicidal ideation and attempts. Total IQ also correlated negatively with several suicidal ideation and behavior subscales.

**Conclusions:**

Adolescents with suicidal concern display distinct cognitive vulnerabilities, particularly in WMI and PRI. These deficits may impair problem-solving, cognitive flexibility, and regulation of intrusive thoughts, thereby increasing vulnerability to suicidal ideation and behaviors. Cognitive screening, combined with psychosocial assessment, may enhance risk stratification and guide early interventions. Bearing in mind these findings, it is essential to view suicidal concern and cognitive vulnerabilities as a bidirectional relationship. Given the cross-sectional design of the study and the possibility that cognitive deficits may also arise because of sustained psychological distress future longitudinal research is needed to clarify directionality and further investigate these associations.

**Disclosure of Interest:**

None Declared

